# Community financing for sustainable food and farming: a proximity perspective

**DOI:** 10.1007/s10460-022-10304-7

**Published:** 2022-03-22

**Authors:** Gerlinde Behrendt, Sarah Peter, Simone Sterly, Anna Maria Häring

**Affiliations:** 1 Unit Policy and Markets in the Agro-Food Sector, Faculty of Landscape Management and Nature Conservation, Eberswalde University for Sustainable Development, Schicklerstraße 5, 16225 Eberswalde, Germany; 2 International Perspectives, Agricultural Policy and Rural Development, Institute for Rural Development Research at Goethe University Frankfurt, Kurfürstenstraße 49, 60486 Frankfurt, Germany

**Keywords:** Proximity, Multiple case study, Crowdfunding, Cooperatives, Citizen shareholder corporations, Profit participation rights

## Abstract

An increasing number of small and medium-sized enterprises (SMEs) in the German organic agri-food sector involves citizens through different community financing models. While such models provide alternative funding sources as well as marketing opportunities to SMEs, they allow private investors to combine their financial and ethical concerns by directly supporting the development of a more sustainable food system. Due to the low level of financial intermediation, community financing is characterized by close relations between investors and investees. Against this background, we apply the proximity concept from economic geography to explore spatial and relational aspects of community financing in the German organic agri-food sector. Based on a qualitative multiple case study approach, we find that the relevance of proximity is twofold. While different forms of proximity between SMEs and their potential investors are key success factors, proximity is also considered as one desired outcome of community financing. Furthermore, our results reveal that the extent to which SMEs rely on particular proximity dimensions distinguishes two different approaches to community financing.

## Introduction

The current agri-food system needs to transform radically as it considerably contributes to the transgression of multiple planetary boundaries (Campbell et al. 2017). Yet, a transformation towards regenerative production practices requires huge investments (FAO 2018; Rockström et al. 2020). In this regard, mobilizing private capital is considered to be a crucial complement to public funding, in particular for small and medium-sized enterprises (SMEs) (Havemann et al. 2020). With changing consumption patterns being a further prerequisite for global food system transformation (Muller et al. 2017; Rockström et al. 2020), community-based financing models which involve citizens and consumers potentially address both sustainable food production and consumption.

While still a niche phenomenon, an increasing share of farms and firms committed to sustainable food applies community financing: They financially involve citizens, for instance, through crowdfunding (Testa et al. 2020), based on a particular legal form such as a cooperative (Wittman et al. 2017) or by means of intermediary organizations that pool citizens’ capital (Stephens et al. 2019). In a broader sense, community supported agriculture (CSA) and sponsorship can also be regarded as community financing instruments.

For SMEs, community financing can increase financial independence from credit institutions (Oberholtzer 2004 as cited in Opitz et al. 2019) and provides an opportunity to access funding which might otherwise be difficult to obtain. Access to finance can be a key obstacle for new farms (Featherstone et al. 2005; EIB 2019; Carlisle et al. 2019), SMEs involved in collaborative short food supply chains (Kneafsey et al. 2015) and green start-ups that offer innovative products or services and/or lack business knowledge (Bergset 2018). Unlike banks and other traditional investors, community investors appear to be more willing to accept the lack of collaterals and low financial returns (Stephens et al. 2019; Partzsch 2019). Apart from financial considerations, community financing can also serve as a marketing tool, helping to build or intensify customer relationships (Brown et al. 2017; Wenz et al. 2018; Schäfer 2019). Yet, public exposure associated with particular community financing models does not attract any capital-seeking SME (Kragt et al. 2021). Furthermore, given the large number of individual investors which need to be managed, community financing often involves huge administration and communication efforts (Agrawal et al. 2014; Messeni Petruzzelli et al. 2019; Wenz et al. 2018).

While CSA has been investigated for more than 20 years (Brown and Miller 2008), very few studies explicitly analyze other community financing models in the agri-food sector. Yet, research from North America (Jayashankar et al. 2015; Stephens et al. 2019) and Germany (Wenz et al. 2018) provides first evidence of an increased spatial and social closeness between investors and investees.

In this regard, the proximity concept from economic geography (Boschma 2005; Torre and Rallet 2005) presents an appropriate analytical framework to further examine spatial and relational aspects of community financing. Research on alternative food networks (AFNs) (Aubry and Kebir 2013; Buclet and Lazarevic 2015; Dubois 2018, 2019; Edelmann et al. 2020; Gugerell and Penker 2020; Gugerell et al. 2021) as well as entrepreneurial and SME finance literature (e.g. Agrawal 2015; Herrmann and Avdeitchikova 2016; Flögel 2018) provide insights into the proximity of food producers to consumers on the one hand and the role of proximity in accessing finance on the other hand. In the context of community financing, however, the relevance of proximity relations between SMEs and their community investors has not yet been examined. With this paper, we address this research gap by using the example of organic food and farming in Germany. Our analysis is guided by an exploratory research question: What kinds of proximity characterize the relationship between SMEs and their community investors in the German organic agri-food sector?

The remainder of the paper is structured as follows. First, we introduce the proximity concept and review literature from related scientific fields which provides empirical evidence concerning the role of proximity. Next, we present the case study design and describe our results. Finally, we conclude with a discussion about the main findings and their implications for further research.

## Literature review

### Multidimensionality of proximity

With its proximity concept, economic geography provides an analytical framework which is primarily applied in the context of regional development, inter-organizational cooperation as well as knowledge transmission and innovation research (Knoben and Oerlemans 2006). Yet, it is increasingly used in other scientific fields (Balland et al. 2015).

There is a general consensus that proximity refers to *being close* and comprises both geographical and non-geographical dimensions (Torre and Rallet 2005; Boschma 2005; Balland et al. 2015). Depending on the scientific field and the level of analysis, however, scholars have developed various ways of conceptualizing different dimensions of proximity (Knoben and Oerlemans 2006). In this regard, we follow Boschma’s (2005) widespread classification which divides proximity into geographical, institutional, cognitive, social and organizational dimensions:

Geographical proximity refers to the “spatial or physical distance between economic actors” (Boschma 2005, p. 69) and represents what is most commonly understood as proximity (Knoben and Oerlemans 2006). While it can be expressed in continuous terms (e.g. in kilometers), the main idea of analyzing geographical proximity is binary: it aims to assess whether two units are “far from” or “close to” each other, which makes it highly relative (Torre and Rallet 2005, p. 49). Being geographically proximate facilitates face-to-face interaction (Boschma 2005), although Torre (2008) argues that permanent geographical proximity is not necessary since the mobility of individuals allows for occasional face-to-face contact. Accordingly, temporary geographical proximity in combination with information and communication technologies (ICTs) can substitute the permanent co-location of actors.

The non-geographical dimensions of proximity either follow a logic of similarity or a logic of belonging (Torre and Rallet 2005; Balland et al. 2013): Actors are considered being close because they share similarities with regard to their knowledge, norms and values etc. or because they belong to the same group, (social) network or organization.

The institutional dimension of proximity covers the “institutional framework at the macro-level” (Boschma 2005, p. 67): being institutionally proximate can involve common formal institutions such as laws and rules but also shared informal institutions like cultural norms, habits and values. A common institutional setting is thought to facilitate economic coordination among actors (Boschma 2005).

Cognitive proximity implies that “people shar[e] the same knowledge base and expertise” (Boschma 2005, p. 63) which facilitates communication and understanding (Nooteboom 1999). Dynamics of cognitive proximity can be understood as learning processes (Balland et al. 2015).

Social proximity refers to trust-based, personal relations between individuals (Boschma 2005). The idea emerges from the embeddedness literature which argues that economic behavior is embedded in social relations and that these relations provide a necessary but not sufficient condition for trust (Granovetter 1985).

Organizational proximity describes “the extent to which relations are shared in an organizational arrangement, either within or between organizations” and potentially reduces uncertainties and opportunism through strong control mechanisms (Boschma 2005, p. 65). This dimension is strongly related to the logic of belonging (Torre and Rallet 2005): Being member of the same organization or network results in a certain degree of organizational proximity between actors.

The different dimensions are strongly interconnected since, on the one hand, a particular form of proximity may favor other proximity relations. For instance, geographical proximity facilitates face-to-face interactions and is therefore likely to foster social proximity (Boschma 2005; Nilsson 2019). On the other hand, one kind of proximity may also compensate for the lack of another proximity dimension. For example, strong ties based on an organizational arrangement may substitute social proximity (Boschma 2005).

### Proximity in alternative food networks

Proximity is a vital concept in the study of AFNs which mainly refer to alternative modes of food production or provisioning such as farmers’ markets or CSA. In their literature review, Forssell and Lankoski (2015) identify three elements of reduced distance between food producers and consumers as core characteristics of AFN: first, they refer to a reduced physical distance. AFNs are usually tied to geographical proximity which is reflected in the notions of local food (Tregear 2011; Eriksen 2013; Carroll and Fahy 2015) and short food supply chains (Kebir and Torre 2013). In this regard, AFN scholars also address temporary geographical proximity: face-to-face interactions at farmers’ markets or trade fairs are likely to compensate for permanent co-location in remote areas (Dubois 2018) or in global value chains (Edelmann et al. 2020). While geographical proximity, whether permanent or temporary, appears to be important for the establishment of personal relations and trust (Milestad et al. 2010), Hinrichs (2000, p. 301) advises not to conflate “spatial relations with social relations”. Second, Forssell and Lankoski (2015) reveal a reduced informational distance, which provides a clear link to cognitive proximity. It relates to the way information about food, its producers and production methods is communicated with consumers in AFNs. Consumers’ knowledge and awareness of food production increases through face-to-face interactions (Dubois 2018; Gugerell and Penker 2020) or through products “embedded with value-laden information” (Renting et al. 2003, p. 400). Such learning potentially increases consumers’ willingness to change behavior or to pay adequate prices (Doernberg et al. 2016; Zoll et al. 2018). Lastly, Forssell and Lankoski (2015) identify a reduced value chain distance which covers the extent of intermediation in the supply chain. Generally, the shorter distance is considered to result in strong (social) relationships between consumers and producers (Forssell and Lankoski 2015). Thus, social proximity is regarded as one key outcome characteristic of AFNs.

Forssell and Lankoski’s (2015) review mainly covers the geographical, social and cognitive dimensions of proximity. Institutional and organizational proximity are much less discussed in AFN literature. Institutional proximity is associated with shared values on environmental protection, organic food production, animal welfare or more generally on prevailing food system structures (Aubry and Kebir 2013; Buclet and Lazarevic 2015; Dubois 2018; Gugerell et al. 2021) but also relates to quality norms and certifications (Edelmann et al. 2020). Organizational proximity considers how producers organize economic coordination (Dubois 2018) or organizational agreements in value chains (Edelmann et al. 2020).

### Proximity in financial markets

Research on entrepreneurial and SME finance also provides insights into the relevance of proximity. Generally, strong evidence exists that financial markets are characterized by a local bias, which describes a preference for spatially proximate investments. For instance, bank lending traditionally serves local markets since transportation cost and information asymmetry increase efforts to establish lending relations at a greater distance (Cerqueiro et al. 2009). Despite technological advancements, geographical proximity still matters in small business lending (Brevoort and Wolken 2009; Agarwal and Hauswald 2010; Flögel 2018). Similarly, spatial effects on investment decisions are demonstrated by the portfolio decisions of socially responsible investment funds (Chen and Nainggolan 2018) and in the context of start-up finance such as venture capital investments (Martin et al. 2005; Cumming and Dai 2010; Lutz et al. 2013) or business angel financing (Herrmann and Avdeitchikova 2016). Yet, it is often not geographical proximity per se that has an impact. It rather provides the basis for other proximity dimensions (Herrmann and Avdeitchikova 2016). For instance, face-to-face interactions between the loan officer and the capital acquirer provide access to soft information (Agarwal and Hauswald 2010). This process is reflected in the notion of relationship lending (Berger and Udell 2006). It refers to a lending technology between banks and SMEs which is based on a direct contact. It is also used to describe lending relationships in social finance (Périlleux 2015) and community financing in the food sector (Stephens et al. 2019). In this context, a low level of financial intermediation facilitates social proximity between investors and investees.

While alternative, ICT-based financing models such as crowdfunding claim to democratize finance and to overcome the local bias, empirical evidence is not so clear. Generally, spatial concentration of investors is found in equity-based crowdfunding (Hornuf and Schmitt 2017; Guenther et al. 2018), reward-based crowdfunding (Mollick 2014; Gallemore et al. 2019) and peer-to-peer lending (Lin and Viswanathan 2016). Particularly investors in food crowdfunding demonstrate a strong bias towards projects in their home countries (Guo et al. 2018). Recent evidence on crowdfunding of climate measures in agriculture also suggests that consumers have a higher willingness to pay for local measures (Stoknes et al. 2021). Furthermore, geographical proximity appears to be particularly relevant in the early stage of a crowdfunding campaign (Agrawal 2015) and when explicitly local projects seek finance (Josefy et al. 2017). However, spatial effects in crowdfunding are also rooted in social networks. Agrawal (2015, p. 271) argues that “what appears to be a geographic distance effect is mostly a social effect”. This clearly corresponds with empirical evidence on the importance of social networks for crowdfunding success (Cai et al. 2019).

A local bias can also be observed among individual investors (Baltzer et al. 2015). Geographical proximity appears to be a significant investment criterion in the slow money movement (Jayashankar et al. 2015) and the community-financed energy sector (Bauwens 2016; Salm et al. 2016; Islar and Busch 2016). This bias towards local investment opportunities is strongly related to investors’ motives, which often go beyond purely financial considerations: Community investors in both the agri-food and energy sector are often willing to accept a low economic return and/or high risks in return for a social or environmental impact (Jayashankar et al. 2015; Islar and Busch 2016; Ebers Broughel and Hampl 2018; Stephens et al. 2019; Partzsch 2019). Since geographical proximity allows them to observe the intended effect of their investment, local impact investment opportunities are considered to be particularly interesting for retail investors (National Advisory Board Germany 2014).

Based on previous research on community financing as well as empirical insights from AFN and finance literature, proximity appears to be central to community financing. However, we assume that the extent to which particular proximity dimensions matter differs widely among the concrete financing models and the level of financial intermediation.

## Material and methods

Case studies allow to examine a contemporary phenomenon thoroughly (Yin 2018). Given the limited research, we chose this approach to explore community financing in the German organic agri-food sector.

Prior to development of the study design, an online research revealed around 600 SMEs,^1^ of which approximately 50% were existing or nascent community-supported farms, 14% applied crowdfunding, 9% used profit participation rights, 4% chose the cooperative model as a funding mechanism and the remaining ones used various models such as direct credits or sponsorship. We further identified 14 operative or nascent intermediary organizations which each cooperated with or provided funding to up to 50 local, organically certified SMEs. While some SMEs exclusively relied on a combination of different forms of community financing, a large share used single community financing instruments to fund a particular project but still drew on more traditional forms of financing.

The results provided evidence for the relevance of the different financing models and established the basis for case selection. In order to cover the variety of community financing models and their concrete implementation, we decided to apply an embedded multiple-case design whereby each case consists of several sub-units of analysis (Yin 2018).

As a first step, we selected community financing models to be considered as cases and investigated in-depth. An increasing relevance in the German organic food sector, yet little scientific evidence on the particular model served as selection criteria. Furthermore, we aimed to consider at least one of the following types of financing models: (1) mere financing instrument, (2) financing model based on a particular legal form and (3) financing in cooperation with an intermediary organization. We selected crowdfunding, profit participation rights, cooperatives and citizen shareholder corporations for our investigations.

Within each of the four case studies, multiple SMEs applying the respective financing model, served as embedded units of analysis. In order to allow for potential differences along the organic food chain, our case studies on crowdfunding, profit participation rights and cooperatives considered at least one SME engaged in one of the following: primary production (agriculture, horticulture or viniculture), food processing and retail or trade. For our case study on citizen shareholder corporations, however, we adopted a slightly different approach: Since we focused on corporations which operate as intermediary organizations and which invest in several SMEs along the organic food chain, we selected two such corporations as our units of analysis. In addition, we exemplarily considered two SMEs which each had obtained funding from one of the respective corporations. The selection of firms was supposed to cover both the typical application of the financing model within the sub-sector and the diversity of usage. Table 1 provides an overview of the number of embedded units of analysis per sub-sector and financing model.

**Table 1:** Number of embedded units of analysis (SMEs and intermediary organizations) per case and sub-sector

Case study	Embedded units of analysis			
	SMEs per sub-sector			Intermediary organization
	Primary production	Food processing	Retail, trade	
Crowdfunding^a^				
SME collects money from a large number of people through an online platform Different types: donation-, reward-, equity- or lending-based^b^	2	2	2	–
Profit participation rights				
SME contractually grants participation in profits and losses	1	2	2	–
Cooperative				
SME financially involves citizens through cooperative shares	1	2	1	–
Citizen shareholder corporation				
SME obtains funding from an intermediary organization which pools citizens’ capital	1	1	–	2

^a^Our analysis focused on SMEs operating in the organic agri-food sector. In the case of crowdfunding, however, a small share of inquired SMEs was not willing to respond to our enquiries and participate in the study. We therefore decided to include two SMEs which had not been certified organic at the time of data collection but offered some of their products in organic quality or had a specific local focus

^b^The four crowdfunding types differ in the return investors receive for their financial contribution. To cover this variety, we selected two SMEs per sub-sector, whereof one used crowdfunding without any monetary reward (donation- or reward-based crowdfunding) while the other offered a monetary return to its supporters (equity- or lending-based crowdfunding)

Case studies typically rely on different sources of evidence (Yin 2018). We decided to draw on document analysis, semi-structured interviews and an online survey. As a first step, we reviewed the websites of the selected SMEs as well as news articles and crowdfunding campaigns. Between September 2018 and July 2019, we conducted 21 semi-structured interviews with representatives of the selected SMEs or intermediary organizations. The interview guide covered questions on the following topics: background information, concrete and legal implementation of the financing model, motives and expectations, relevance in financial terms, communication with investors, commitment of and relationship to investors, challenges and success factors. In order to gain insights into the investors’ perspective, we asked the SME managers to connect us with their investors. We set up a short online survey to obtain an overview of investors’ motives and received 107 valid responses. In addition, we conducted 20 semi-structured interviews with investors in order to ascertain in greater detail why they participated, how they relate to the respective SME and which challenges they face. This paper is mainly based on the insights from the semi-structured interviews which were audio recorded and transcribed.

We applied a mix of deductive and inductive coding to the material by using MAXQDA. The five proximity dimensions as defined by Boschma (2005) served as the deductive starting point for our coding process. In a first step, we read carefully through the material and tried to assign relevant statements to one of the five proximity dimensions. For this purpose, we checked whether interviewees made any spatial reference (geographical proximity), addressed the personal relationship between SME (manager) and investors (social proximity), commented on or compared the knowledge base of SMEs and investors (cognitive proximity), referred to shared formal or informal institutions (institutional proximity) or an organizational setting which ties a SME and its investors (organizational proximity). While it was straightforward to assign statements to geographical and social proximity, interviewees referred rather implicitly to the cognitive, institutional and organizational dimensions of proximity. Coding with respect to the latter proximity dimensions therefore required some interpretation, which was guided by the logics of similarity and belonging (Torre and Rallet 2005): We assigned any information which indicated similarities (or disparities) between SMEs and their (potential) community investors with regard to knowledge or values to cognitive or institutional proximity respectively. Similarly, any reference to membership of a particular organization or network was linked with organizational proximity. During an iterative process, we then built sub-codes and refined our coding framework. Ultimately, we inductively developed four additional categories, which completed our coding framework: proximity as prerequisite for successful community financing (success factors), proximity as a desired outcome of community financing, related difficulties and links between different proximity dimensions.

## Results

### Geographical proximity

The relevance of geographical proximity differs widely between the units of analysis. To a certain extent, it relates to the respective marketing channels: on the one hand, all case studies provide examples of community financing projects where a large share of investors live close to the capital acquiring SME. This applies mainly to owner-managed retail shops as well as farms and artisanal food producers who market their products directly to consumers. Furthermore, citizen shareholder corporations that limit their economic activity to a particular region, mostly address investors from within the defined area. On the other hand, the case studies on crowdfunding, profit participation rights and cooperatives also reveal nationwide participation in community financing projects. This applies to both SMEs which market their products all over Germany and SMEs whose marketing has more of a local focus.

It becomes clear that geographical proximity can—but does not necessarily—provide the basis for successful community financing. Existing SMEs profit from their local customer base since they are likely to become investors, too. However, this is not an expressly spatial effect. Equally important are the familiarity with and the reputation of the SME as well as its network within the region. Furthermore, where permanent geographical proximity is not given, most of the analyzed SMEs rely on temporary geographical proximity to facilitate face-to-face contacts with their (potential) investors. While in the case of crowdfunding, this mainly refers to events organized in order to convince potential investors, SMEs that use profit participation rights or the cooperative model also organize events for the existing investors. In many cases, this goes beyond legally required events such as the general meeting of cooperative members. In the case of citizen shareholder corporations, the annual meeting of shareholders at least provides an opportunity for face-to-face interaction between SME representatives and investors.

While economically viable surroundings can foster community financing, interviewees also emphasize regional specifics that hamper the mobilization of local investors. For instance, one SME owner argues that the anonymity and overstimulation in a large city impedes a successful crowdfunding campaign. In contrast, another interviewee states that it appears to be more difficult to convince people in rural areas to invest. Furthermore, one cooperative manager stresses that local citizens are critical of the cooperative model since a well-known SME which transformed into a cooperative later went bankrupt. Accordingly, local citizens are difficult to persuade. Lastly, one interviewee argues that regional specifics regarding leasing conditions are a barrier to cooperating with an intermediary organization that is active on a national level.

### Social proximity

Similarly to geographical proximity, the role of social relations between SMEs and their investors varies considerably. A large share of the SME managers interviewed state that they know at least some of their investors personally since they come from their personal network, family and friends or the existing customer base. Many investors who were interviewed confirm that personal ties motivate them to participate. Social relations are of particular importance for SMEs that use profit participation rights and cooperative managers who have strong personal ties to the founding and active members. Likewise, the first supporters or investors of crowdfunding campaigns often originate from the social network of SME founders and managers. Depending on the crowdfunding platform’s own network and outreach, however, a major share of investors remains anonymous. In the case of citizen shareholder corporations, relations between shareholders and SMEs are intermediated through the corporation. Accordingly, strong social ties are less common.

Generally, most SME managers consider social proximity and social networks to be crucial success factors for community financing. An existing customer base or other personal networks are characterized by a certain degree of trust which in turn facilitates the acquisition of investors within these networks. Word-of-mouth recommendations and reputation often benefit community financing projects. Furthermore, being present at or organizing events such as guided farm or firm tours facilitates face-to-face interactions with potential investors. It allows direct address and the opportunity to explain the community financing project. Crowdfunding, much more so than other models, relies on existing social media or newsletter networks to approach potential investors. However, the case study on crowdfunding also provides the only examples whereby the personal network of SME managers is nearly irrelevant.

One farm manager argues that small investors with whom they establish a trustful relationship are more patient than their bank which is regarded to be an advantage of social proximity. However, a large share of SME managers interviewed emphasize that establishing and maintaining relations to a large number of investors is extremely time-consuming. One SME owner feels that the personal relationship to investors increases the pressure on him to succeed. Lastly, as two founders argue, social proximity does not guarantee successful community financing, either because the personal network lacks commitment or because SME managers refrain from asking their social network for financial support.

Most SMEs use various channels to stay in touch with their investors, communicating via social media, phone or email and organizing regular events. While crowdfunding is characterized by rather little contact between SMEs and existing investors, SMEs that use financing models based on long-term investments often establish strong relationships with their investors.

This aspect relates to one important idea of community financing: SMEs do not only use it as a financing instrument, instead they view it as a marketing tool, e.g. to build and strengthen customer relationships. Accordingly, social proximity is not only a success factor but also one aim of community financing. This motivation is specifically stated in the context of profit participation rights. Other interviewees do not explicitly articulate this aim. However, they appreciate the positive impact of the community financing project on their customer base and sales.

### Cognitive proximity

SME managers rarely address the cognitive proximity to their community of investors. Nonetheless, it contributes to the success of community financing projects. One interviewee argues that people who have prior knowledge of sustainable food and farming are easier to convince to make a financial contribution. Furthermore, several SME managers emphasize that the produce, topic or investment object needs to be easy to understand and attractive for potential investors. The more vivid and emotional the investment object, the easier it is to convince potential investors to participate. Events such as guided tours or tastings allow for the direct observation of food production or processing and give a better understanding of the investment project. While this example refers to the acquisition of investors, transparent food production and processing is also regarded as a desired outcome of community financing.

Few interviewees describe a cognitive distance to their investors. They argue that small investors often lack financial or legal knowledge. Unlike banks, they can usually neither evaluate the economic state of the SME nor the risk associated with their investment. This is considered to result in a particular responsibility of SMEs towards their investors.

### Institutional proximity

Institutional proximity is not explicitly addressed by interviewees. However, both interviews with SME managers and investors provide evidence that a share of investors neither have any social connections to the SME nor do they live geographically close. Their motivation to contribute financially relates to notions of organic food, support of smallholder and artisanal food producers as well as ethical and transparent investments. They participate because they share particular values with the capital acquiring SME or are convinced of investment criteria established by intermediary organizations (e.g. organic certification as minimum requirement). In contrast, financial motives are hardly mentioned by community investors. Several examples from the case studies on crowdfunding, cooperatives and citizen shareholder corporations also reveal that an institutional distance can impede the success of community financing. Potential investors sense that a crowdfunding platform or the legal form does not correspond with their values. For instance, many potential investors regard corporations as the embodiment of an economic system they do not want to support and this discourages them from participating. Hence, proximity with regard to informal institutions is an important success factor for community financing.

### Organizational proximity

Organizational proximity between SMEs and investors matters where an organizational structure provides the basis for their (indirect) relationship. For instance, shareholders and SME managers are organized under the umbrella of a citizen shareholder corporation even if they do not share personal ties. A side effect of this is that SMEs also connect with each other. Similarly, cooperating with a crowdfunding platform, which is linked to a large network of investors, can ensure the success of a crowdfunding campaign. Accordingly, membership of or links to existing organizations and networks can be important success factors for community financing. However, one interviewee states that a geographical focus of such organizations increases the willingness of citizens to participate. While it was not explicitly mentioned in the case study on citizen shareholder corporations, the regional structure of these organizations appears to support this argument.

Organizational proximity is also regarded as one aim of community financing. For instance, as investors become members of a cooperative or holders of profit participation rights, their sense of belonging increases. Their identification with and commitment to the SME can result in increased and more regular sales.

## Discussion and conclusion

Generally, our findings reveal that the role of proximity in community financing is twofold (see Table 2). On the one hand, proximity between SMEs and their (potential) investors is a key success factor. On the other hand, a large share of SMEs also considers proximity as one desired outcome of community financing. In the following, we discuss the two aspects separately.

**Table 2:** Relevance of different proximity dimensions in community financing

	Geographical proximity	Social proximity	Cognitive proximity	Institutional proximity	Organizational proximity
Proximity as…					
…success factor of community financing	Local customer base Familiarity and reputation within the region Temporary geographical proximity through organization of events High-income region	Direct customer contact Trust based on social relations Large social network, family and friends Reputational inference	Vividness of an investment object Events: farm or firm visits, tastings Awareness of food-related sustainability among potential investors	Shared values and vision on food system and/or economic system	Membership of existing organizations or networks Regional focus of an organization
…desired outcome of community financing	–	Building and strengthening customer relations	Establishing transparency	–	Increasing commitment and identification
Related difficulties	Regional specifics	Time-consuming relationship management Lack of commitment in personal network Timidity when asking for money Responsibility for acquaintances’ capital	Economic, financial and legal illiteracy of small investors	Values apparently inconsistent with particular legal form	–
Related SME types and financing models	SMEs that market their products directly to local customers Temporary geographical proximity to connect with potential investors (all models) and to keep in touch with existing investors (all models but crowdfunding)	SMEs that (aim to) market their products directly Least relevant in crowdfunding	Relevant for all SME types and financing models	SMEs that do not market their products directly	Models based on indirect relations through intermediary organizations or platforms Cooperative model
Links to other dimensions	Can provide the basis for social proximity	Can provide the basis for cognitive proximity		Can compensate for a lack of social and/or geographical proximity	Can compensate for a lack of social and/or geographical proximity Can complement geographical and/or institutional proximity

### Proximity as a key success factor

Each of the five proximity dimensions can contribute to successful community financing. First of all, the effects of geographical, social and cognitive proximity appear to be strongly interconnected. Geographical proximity, whether permanent or temporary, facilitates direct contact between SME managers and potential investors and can provide the basis for personal ties. Many investors in our study argue that a personal, trust-based relation to the respective SME is a major reason for their investment. Trust is the key dimension of social proximity (Boschma 2005) and is one possible explanation why investors decide to make a financial contribution even if it is relatively risky. Stephens et al. (2019) provide one example where personal relations are considered a more effective mechanism to reduce risks than collateral-based lending. Furthermore, temporary geographical proximity and face-to-face interactions during farm or firm visits allow potential investors to observe production processes and have the project explained to them in person. Thus, their awareness of the SME’s financial needs and sustainable food and farming increases as does their willingness to contribute financially. It can therefore be said that geographical proximity indirectly affects the success of community financing since it can be the basis for strong personal ties, which in turn can reduce cognitive distance between SME owners and potential investors. However, even if various examples in the AFN and finance literature reveal close links between geographical and social proximity (Milestad et al. 2010; Agrawal 2015; Herrmann and Avdeitchikova 2016), it is important to understand that geographical proximity does not necessarily lead to social proximity and trust between actors (Nilsson 2019).

Institutional and organizational proximity can also be relevant success factors, particularly when social and geographical proximity are lacking. On the one hand, our results reveal that some investors support SMEs which are located far away and to which they do not have any personal ties. Their main motivation lies in specific values that they share with the respective SME. They aim to support a distinct kind of food production such as organic, small-scale or artisanal. Accordingly, institutional proximity can benefit community financing if the SME succeeds in clearly communicating its values to potential investors. While not mentioned by any interviewed investors as being crucial for their investment, organic certification can provide a more formal way of demonstrating compliance with particular standards (Dubois 2019; Edelmann et al. 2020). For instance, being organically certified is a minimum requirement for SMEs to obtain funding from the citizen shareholder corporations included in our case study. Such an investment criterion can be considered as a formal institution corresponding with investors’ preferences and reflecting their institutional proximity. On the other hand, organizational proximity can also facilitate community financing and provides an opportunity for SMEs that lack a large social network. It mainly refers to financing models where an organization or online platform mediates relations to an existing network of potential investors. The network members often share certain values which provides a clear link to institutional proximity and diminishes the relevance of geographical proximity. However, examples from our case studies also indicate that regionally bound organizations or networks can be more effective in attracting new investors.

### Proximity as a desired outcome

While proximity generally favors community financing, many of the SME managers interviewed further regard social, organizational and cognitive proximity as desired outcomes of community financing. First of all, they consider community financing to be a marketing instrument which allows them to establish and foster customer relations. Wenz et al. (2018) confirm that these financing models often aim to build strong ties which in turn leads to trust between customer-investor and SME. Marketing considerations, however, are also linked to the organizational dimension of proximity. Irrespective of any personal ties, being member of an organization (e.g. citizen shareholder corporation or cooperative) can increase a sense of belonging. A few SME managers argue that these investors tend to support *their* SMEs and contribute to higher sales. Lastly, one farm manager argues that community financing enables her to give investors a better understanding of sustainable food production and thus decreases the cognitive distance.

### Two main approaches to community financing

Generally, we observe two main approaches to community financing. Irrespective of the sub-sector, SMEs tend to rely either on (a) a combination of geographical, social and cognitive proximity or (b) on institutional and organizational forms of proximity. Direct interaction and reputational inference based on social proximity can increase trust between actors (Nilsson 2019) and, as argued in AFN literature, facilitates communication of quality features and makes more formal institutions such as labels and certifications unnecessary (Renting et al. 2003; Schäfer 2019; Dubois 2019). Establishing and maintaining direct relations with a large number of investors is, however, very time-consuming and often represents an additional burden to SMEs which still rely on more traditional financing sources. Furthermore, close personal ties to investors can also be detrimental in the sense that SME owners feel under pressure. Instead, by analogy with certification schemes in AFNs which are associated with organizational and institutional proximities (Dubois 2019), community financing models characterized by a higher level of financial intermediation can diminish required efforts by and complexity for SMEs. This, in turn, decreases social proximity and the potential marketing effect of community financing. SME managers therefore need to consider thoroughly which of the two approaches to community financing better fits their idea and motives.

Yet, while the cooperation with intermediary organizations and crowdfunding appear to rely more on institutional and organizational proximity, boundaries between the two approaches become blurred and one cannot clearly assign a specific financing model to one of them. In the case of crowdfunding, for instance, we observe both one SME which relies almost entirely on the strong network of the online platform and one SME which uses a platform to comply with regulations but attracts mostly investors from its existing customer base. Accordingly, the approach to community financing rather depends on the concrete implementation than on the particular financing models.

To a certain extent, both approaches to community financing are characterized by different forms of local biases. On the one hand, corresponding with evidence from the finance and banking literature, geographical proximity has an indirect impact because it correlates with and mediates other forms of proximity (Agrawal 2015; Herrmann and Avdeitchikova 2016). On the other hand, a preference for local investments can also be observed where SMEs rely mainly on institutional and organizational proximity. While these proximity dimensions are thought to potentially compensate for a lack of co-location, shared values with regard to local food systems explain a second form of local bias.

## Concluding remarks and outlook

With this paper, we aimed to investigate the phenomenon of community financing for sustainable food and farming through the lens of proximity. While AFN scholars already analyze different proximity dimensions between food producers and consumers, previous research on financial markets does not explicitly consider the multidimensional proximity concept and mostly refers to proximity as a spatial dimension. In this regard, our study provides a novel link between economic geography, AFN literature and SME finance.

Our embedded multiple-case design helps to deepen the understanding of proximity relations between SMEs and their community investors. It provides a systematization that is not only of academic interest but also of practical relevance: Those who use or design community financing instruments can particularly build on the distinction between the two main approaches we identified in our study. A reflection on appropriate financing models is supposed to benefit from an understanding of how different forms of proximity relate to each other and which trade-offs exist.

Yet, it is important to acknowledge that our findings reflect a pre-pandemic world. While previous research in the context of food and farming indicates that ICT can support but not replace other forms of proximity (Townsend et al. 2016; Bos and Owen 2016), the role of “virtual proximity” requires further investigation (Dubois 2018, p. 11). Given that the COVID-19 pandemic has considerably accelerated the adoption of digital solutions in the agri-food sector (Nemes et al. 2021; Apostolopoulos et al. 2021), future research could focus on how an increased use of ICT affects proximity relations between SMEs and their community investors.

In conclusion, community financing can be considered as a clear niche in relation to institutional investors or impact investing organizations which provide private capital for more sustainable food systems (GIIN 2020; Havemann et al. 2020). Yet, transition research acknowledges the importance of innovations emerging from niches (Loorbach et al. 2017; El Bilali 2020) and recent research from the German agri-food sector attributes a high transformative potential to particular community financing models (Haack et al. 2020). In our view, its potential to increase investors’ awareness towards sustainable consumption—analogous to rising consumer awareness in AFNs (Forssell and Lankoski 2015)—is particularly worth further examination. However, we assume that both rather unfavorable risk-return characteristics for potential investors and huge administration and communication efforts for SMEs are key barriers to an increasing relevance of community financing. Future research should therefore address the appropriateness of supportive policy frameworks or measures (e.g. tax incentives to decrease investors’ risk, as described by Stephens et al. (2019)) and analyze financing structures which facilitate the incorporation of community investments in terms of blending different capital sources for more sustainable food systems.

## Data Availability

Not applicable.

## Abbreviations

AFN: Alternative food network
CSA: Community supported agriculture
ICT: Information and communication technology
SME: Small and medium-sized enterprise

## Footnotes

1 The results of the online research do not raise any claim to completeness. Yet, the number of identified SMEs indicates the niche character of community financing in the German organic food sector which consists of more than 30,000 organic farms (in relation to 260,000 farms and agricultural holdings in total), more than 11,000 processing firms and more than 3500 firms involved in trade and retail of organic products (Federal Office for Agriculture and Food 2021; Federal Statistical Office 2021).
